# Genetics, Host Range, and Molecular and Pathogenic Characterization of *Verticillium dahliae* From Sunflower Reveal Two Differentiated Groups in Europe

**DOI:** 10.3389/fpls.2018.00288

**Published:** 2018-03-09

**Authors:** Alberto Martín-Sanz, Sandra Rueda, Ana B. García-Carneros, Sara González-Fernández, Pedro Miranda-Fuentes, Sandra Castuera-Santacruz, Leire Molinero-Ruiz

**Affiliations:** ^1^Pioneer Hi-Bred International, Inc., La Rinconada, Spain; ^2^Department of Crop Protection, Institute for Sustainable Agriculture, Spanish National Research Council, Córdoba, Spain

**Keywords:** control strategies, crop rotation, genetic resistance, molecular markers, races of *V. dahliae*, pathotypes of *V. dahliae*, soilborne fungus

## Abstract

Verticillium wilt and leaf mottle of sunflower, caused by the fungus *Verticillium dahliae* (*Vd*) has become a major constraint to sunflower oil production in temperate European countries. Information about *Vd* from sunflower is very scarce despite genetics, molecular traits and pathogenic abilities of fungal strains affecting many other crops being widely known. Understanding and characterizing the diversity of *Vd* populations in those countries where sunflowers are frequent and severely affected by the fungus are essential for efficient breeding for resistance. In this study, we have analyzed genetic, molecular and pathogenic traits of *Vd* isolates affecting sunflower in European countries. When their genetics was investigated, almost all the isolates from France, Italy, Spain, Argentina, and Ukraine were assigned to vegetative compatibility group (VCG) 2B. In Bulgaria, Turkey, Romania, and Ukraine, some isolates were assigned to VCG6, but some others could not be assigned to any VCG. Genotyping markers used for *Vd* affecting crops other than sunflower showed that all the isolates were molecularly identified as race 2 and that markers of defoliating (D) and non-defoliating (ND) pathotypes distinguished two well-differentiated clusters, one (E) grouping those isolates from Eastern Europe and the other (W) all those from the Western Europe and Argentina. All the isolates in cluster W were VCG2B, while the isolates in cluster E belonged to an unknown VCG or to VCG6. When the host range was investigated in the greenhouse, the fungus was highly pathogenic to artichoke, showing the importance of farming alternatives in the management of Verticillium attacks. Sunflower genotypes were inoculated with a selection of isolates in two experiments. Two groups were identified, one including the isolates from Western Europe, Argentina, and Ukraine, and the other including isolates from Bulgaria, Romania, and Turkey. Three pathogenic races were differentiated: V1, V2-EE (Eastern Europe) and V2-WE (Western Europe). Similarly, three differentials are proposed for race identification: HA 458 (universal susceptible), HA 89 (resistant to V2-EE, susceptible to V2-WE) and INRA2603 (susceptible to V2-EE, resistant to V2-WE). The diversity found in *Vd* affecting sunflower must be taken into account in the search for resistance to the pathogen for European environments of sunflower production.

## Introduction

Verticillium wilt and leaf mottle (VWLM), caused by the fungus *Verticillium dahliae* Kleb. (*Vd*), has traditionally been a major disease of sunflower in Argentina and the United States (Gulya et al., 1997; Radi and Gulya, 2007; Galella et al., 2012) as well as in temperate European countries (Harveson and Markell, 2016). However, disease incidence in France, Italy, Spain, and countries around the Black Sea has dramatically increased in recent years and in some regions, like southern France, it is becoming a major constraint to sunflower oil production (Harveson and Markell, 2016; Debaeke et al., 2017). *Verticilliun dahliae* is a soilborne ascomycete with a wide range of host crops. Besides sunflower, it causes important yield losses in artichoke (*Cynara cardunculus* L. var. *scolymus*), cauliflower (*Brassica oleracea* var. *botrytis* L.), cotton (*Gossypium hirsutum* L.), eggplant (*Solanum melongena* L.), lettuce (*Lactuca sativa* L.), olive tree (*Olea europaea* L.), potato (*Solanum tuberosum* L.), tobacco (*Nicotiana tabacum* L.), and tomato (*Solanum lycopersicum* L.), among others (Pegg and Brady, 2002). In Spain, Verticillium constitutes an important constraint for the production of cotton, artichoke, and, particularly, of olive tree (Bejarano-Alcázar et al., 1996; Berbegal et al., 2010; Jiménez-Díaz et al., 2011; López-Escudero and Mercado-Blanco, 2011). Also in Spain, VWLM outbreaks have repeatedly been observed in the last few years in sunflower fields of Cadiz province (García-Ruiz et al., 2014), where it is grown in alternation with other crops, particularly cotton and/or tomato. Some fields have in fact even been turned into olive tree groves. Host specialization occurs in *Vd*, meaning that isolates from a given host may be pathogenic on other hosts but, generally, they are more virulent (symptoms are more severe) on the hosts from which they were obtained. In some *Vd* isolates, host specialization is more pronounced (Bhat and Subbarao, 1999; Douhan and Johnson, 2001). In areas where sunflower is grown in alternation with other crop species, determination of the host range specificity of *Vd* affecting sunflowers is important for the correct management of the whole cropping system.

Clonality in *Vd* is described by means of vegetative compatibility, which refers to the genetically controlled ability of individual fungal strains to undergo hyphal anastomosis and form stable heterokaryons. Vegetatively compatible isolates are placed in the same vegetative compatibility group (VCG). In spite of there being many studies on the genetic diversity of *Vd* from artichoke (Jiménez-Díaz et al., 2006), cotton (Dervis et al., 2008; Korolev et al., 2008), eggplant (Dervis et al., 2009b), olive tree (Navas-Cortés et al., 2009; Dervis et al., 2010), potato and mint (Dung et al., 2013) or sugarbeet (Strausbaugh et al., 2016) among other crops, the genetic characterization of *Vd* isolates from sunflower has been scarcely addressed. Isolates of *Vd* affecting sunflowers in Canada showed weak reactions with testers from VCG4A and 4B; one isolate was identified as VCG3 and another one was compatible with all VCG groups except VCG2A (El-Bebany et al., 2013). In previous works by our research group, isolates from Argentina and Spain were adscribed to VCG2B (García-Carneros et al., 2014). Since *Vd* reproduces asexually, isolates in the same VCG could be genetically distinct populations with a similarity in a number of physiological, ecological, pathogenic, and host-range traits (Jiménez-Díaz et al., 2006; Collado-Romero et al., 2008; Korolev et al., 2009). Thus, genetic diversity of *Vd* isolates from sunflower could be intimately associated with disease occurrence and severity as a consequence of particular interactions *Vd* isolate – sunflower genotype.

In some crops, such as cotton or olive tree, isolates of *Vd* causing infection are pathogenically characterized by assignment to defoliating (D) or non-defoliating (ND) pathotypes, which are identified on the basis of their capacity to cause, or not, the complete fall of green leaves (Rodríguez-Jurado et al., 1993; Bejarano-Alcázar et al., 1996). Molecular analyses using SCAR markers differentiated a genetically homogeneous group of D isolates belonging to VCG1A (Mercado-Blanco et al., 2002). In contrast, these markers showed a high molecular diversity of ND pathotypes belonging to 2A, 2B, and 4B VCGs (Pérez-Artés et al., 2000; Mercado-Blanco et al., 2001, 2002, 2003). Preliminary results from our research group showed that the molecular pattern of *Vd* isolates infecting sunflower in Argentina and Spain matched that of the ND pathotype of artichoke and/or cotton, pointing to the closeness between ND isolates affecting these three crops and, therefore, suggesting that any of them could serve as a carrier and source of inoculum for Verticillium outbreaks (García-Carneros et al., 2014).

While isolates of *Vd* infecting crops like cotton and olive tree are assigned to D or ND pathotypes, races of *Vd* pathogenic to tomato, lettuce, and sunflower are distinguished depending on the genes of resistance that they overcome. Race 1 and race 2 have been described for isolates of *Vd* pathogenic to tomato (Alexander, 1962) and lettuce (Vallad et al., 2006; Hayes et al., 2007). Moreover, the races of *Vd* on tomato and lettuce have been reported as being correlated (Maruthachalam et al., 2010). Sequence similarity between the resistance gene of lettuce (*Vr1*) and that of tomato (*Ve1*) suggests that they share similar race 1-specific genes for resistance to *Vd* (Hayes et al., 2011). Also, race 1 is characterized by the presence of the effector gene *Ave1*, conferring avirulence to lettuce or tomato that carry the resistance genes *Vr1* or *Ve1*, respectively (de Jonge et al., 2012). Conversely, race 2 *Vd* isolates lack *Ave1* and they are, therefore, potentially virulent on plants carrying resistance to race 1 (de Jonge et al., 2012; Short et al., 2014). Race 1 seems to have arisen once by horizontal gene transfer and is genetically much less diverse than race 2 (de Jonge et al., 2012; Jiménez-Díaz et al., 2017). Race 2 occurs worldwide and it causes disease on cultivars from a range of crops for which effective resistance has not been reported (Maruthachalam et al., 2010; Short et al., 2014; Sandoya et al., 2017). Regarding *Vd* on sunflower, the first race (NA-1) was detected in the United States, and it was controlled by the resistance in HA 89, which was associated to a single major gene (Fick and Zimmer, 1974; Gulya et al., 1997). New races overcoming this resistance, and apparently different to each other, have been reported later on the basis of phenotypic characterization: one (NA-Vd2) in the United States (Gulya, 2007), four in Argentina (Bertero de Romano and Vázquez, 1982; Galella et al., 2004; Clemente et al., 2017) and one in Spain (García-Ruiz et al., 2014). No comparative studies of these proposed races of *Vd* affecting sunflower have so far been conducted, and neither are any relationships with races 1 and 2 on tomato and lettuce known.

From a practical point of view, genetic resistance has been the most effective method for controlling VWLM in sunflower for nearly 50 years. Initial sources of resistance were identified in Canada in the 1950s (Putt, 1958). The inheritance of resistance in some inbred lines was found to be qualitative or of a complete dominance and designated as *V_1_* (Putt, 1964). The same type of resistance was found 10 years later in certain inbred lines from the USDA collection, such as HA 89 (Fick and Zimmer, 1974), which is a recurrent parent in the development of resistant hybrids, particularly in public sunflower breeding programs. The new races of *Vd* are not controlled by the resistance in HA 89 (see above). Instead, some of them seem to be controlled by the resistance of some entries of the USDA sunflower collection, such as PI507901 (Radi and Gulya, 2007) or the inbred lines HA 300 and HA 371 (Gulya et al., 1997). Moreover, the inheritance of resistance to race NA-Vd2 appears to be recessive or additive in some lines, and the breeding alternative of pyramiding quantitative resistance is being explored in Argentina (Galella et al., 2012). Frequent outbreaks of VWLM in sunflower-growing countries suggest that the resistance in commercial hybrids was overcome by the pathogen and this makes it urgent to identify plant material which could serve as a donor of resistance against the current races of the fungus worldwide.

This work was conceived from a holistic perspective since a bewildering amount of scientific information is available for *Vd* affecting many crops but *Vd* being pathogenic on sunflower is largely unknown. Here we describe the population structure of the *Vd* affecting sunflowers in countries of Europe where VWLM recurrently threatens oil production: Bulgaria, France, Italy, Romania, Spain, Ukraine, and Turkey. Genetic, molecular and pathogenic traits of the fungal collection were studied and, because of its epidemiological significance, we also addressed to what extent *Vd* from sunflower can be pathogenic on other crops.

## Materials and Methods

### Isolates of *Verticillium dahliae* From Sunflower

All the isolates of the fungus were recovered from affected sunflowers that were collected between 2009 and 2016 in Argentina, Bulgaria, France, Italy, Romania, Spain, Turkey, and Ukraine. Because of the importance Verticillium wilt has in sunflowers in Argentina and although we did not have a set of isolates representing the race diversity of *Vd* in that country, one isolate from Argentina was included as representative. The plants showed interveinal chlorosis and yellowing, as well as wilt symptoms (see **Supplementary Figure S1A**). Their reference and information on the year of collection and geographical location of the samples are presented in **Table 1**. Cross sections of the stem base and petiole tissues of all the plants were analyzed. Each section was divided into two–six pieces that were surface-disinfested for 3 min by immersion in 10% household bleach (40 g of active chlorine per liter), rinsed in deionized water for 3 min and air dried using a vertical laminar flow cabinet. Segments 2 to 4 mm long of sunflower tissue were aseptically transferred to petri plates containing potato dextrose agar (PDA). Plates were incubated at 25°C for 72 h in darkness. Colonies were morphologically confirmed by observation under the stereoscope. Only one colony among all those recovered from the same field was selected for further studies, and a minimum of two monoconidial cultures were obtained by the following procedure. Each isolated colony was transferred to PDA and incubated in the laboratory at 25°C. After 8–10 days, the plates were flooded with 5 ml of sterile deionized water each and swirled gently. The conidial suspension was filtered through two layers of sterile gauze. Five serial 1:10 dilutions were prepared from the initial suspension and, from each of them, a small volume was streaked onto Water Agar (WA) medium following a zigzag distribution. Plates were incubated at 28°C for 24 h in darkness. Germinating conidia were then identified under the stereoscope and individually transferred to PDA. The final colonies were confirmed as being *Vd* based on morphological characters, and labeled as monoconidial isolates. Monoconidial and original isolates were stored in PDA as part of the fungal collection of the Laboratory of Field Crop Diseases at the Institute for Sustainable Agriculture, Córdoba, Spain.

**Table 1:** Isolates of *Verticillium dahliae* used in this work, listed by year of collection, geographic origin and host, and genetic characterization by means of assignment to Vegetative Compatibility Group (VCG).

Isolate^∗^	Year of collection	Geographical location (Country, Province)	Host	VCG
VdS0109	2009	Spain, Andalusia	Sunflower	2B
VdS0209	2009	Spain, Andalusia	Sunflower	2B
VdS0112^∗^	2012	Argentina, Balcarce	Sunflower	2B
VdS0212	2012	Spain, Andalusia	Sunflower	2B
VdS0312	2012	Spain, Andalusia	Sunflower	2B
VdS0113^∗a^	2013	Spain, Cádiz	Sunflower	2B
VdS0213	2013	Spain, Cádiz	Sunflower	2B
VdO0913	2013	Spain, Jaen, Sant. Puerto	Olive tree	1A
VdO1113	2013	Spain, Jaen, Cast. Locubin	Olive tree	2A
VdS0114	2014	Turkey, Thrace	Sunflower	–
VdS0214^∗^	2014	Turkey, Thrace	Sunflower	6
VdS0314	2014	Turkey, Thrace	Sunflower	–
VdS0414	2014	Turkey, Thrace	Sunflower	–
VdS0514^∗^	2014	Turkey, Thrace	Sunflower	6
VdS0614	2014	Turkey, Thrace	Sunflower	–
VdS0714^∗^	2014	Bulgaria, Krumovo	Sunflower	6
VdS0814	2014	Bulgaria, Krumovo	Sunflower	–
VdS0914^∗^	2014	Romania, Valu lui Traian	Sunflower	–
VdS1014	2014	Romania, Valu lui Traian	Sunflower	–
VdS1114	2014	Romania, Valu lui Traian	Sunflower	–
VdS1314	2014	Romania, Movila Braila	Sunflower	–
VdS1414^∗^	2014	France, Montgiscard	Sunflower	2B
VdS1514^∗^	2014	France, Montbeilhan, Marignac	Sunflower	–
VdS1614^∗^	2014	France, Vacquiès, Saint-Aignan	Sunflower	2B
VdS1714^∗^	2014	France, Vacquiès, Saint-Aignan	Sunflower	2B
VdS0115	2015	Spain, Cádiz	Sunflower	2B
VdS0215	2015	Spain, Cádiz	Sunflower	2B
VdS0116^∗^	2016	Bulgaria, Dobrich	Sunflower	6
VdS0216^∗^	2016	France, Montech	Sunflower	2B
VdS0316^∗^	2016	Italy, Macerata	Sunflower	2B
VdS0416^∗^	2016	Italy, Pisa	Sunflower	2B
VdS0516^∗^	2016	Romania, Cuocorova	Sunflower	–
VdS0616^∗^	2016	Romania, Slava Rusǎ	Sunflower	–
VdS0716^∗^	2016	Spain, Seville	Sunflower	2B
VdS0816^∗^	2016	Turkey, Edirne	Sunflower	–
VdS0916^∗^	2016	Turkey, Kastamonu, Ahmetbey	Sunflower	6
VdS1016^∗^	2016	Ukraine, Manvelivka, Dnepropet	Sunflower	2B
VdS1116^∗^	2016	Ukraine, Velyki Sorochyntsi, Polta	Sunflower	6

*^*a*^Isolate identified as a new race of Vd overcoming V_*1*_ gene in HA89 by García-Ruiz et al. (2014).*

*^∗^Isolates included in the pathogenic characterization with sunflower genotypes.*

### Genetic Diversity: Determination of Vegetative Compatibility Groups

The VCG of 38 monoconidial isolates, including those previously characterized by our research group (García-Carneros et al., 2014), were determined by generation and characterization of nitrate non-utilizing (*nit*) mutants of each of them and determination of vegetative compatibility. *Nit* mutants were generated on Water Agar Chlorate medium as colonies presenting a faint growth on Czapek-Dox Agar (CDA) with no aerial mycelium (Korolev and Katan, 1997) and phenotyped on CDA amended with hypoxanthine as described by Correll et al. (1987). Complementation tests were done by pairing *nit* mutants of the isolates with the complementary mutants of the international OARDC (The Ohio State University, Wooster, OH, United States) reference testers, Israeli *nit* testers and testers of Washington State University (Pullman WA, United States): T9 isolate (VCG1A), Ep8M and Ep52 isolates (VCG2A), Cot200 and Cot254 isolates (VCG2B), 70-21 isolate (VCG3), 131M isolate (VCG4A), Pt15M isolate (VCG4B), and MT isolate (VCG6). Pairings were done following the methodology of Collado-Romero et al. (2006). Mycelial plugs of *nit* testers and *nit* mutants of test (unknown) isolates were placed 1.5 cm apart on CDA in petri plates and incubated at 25°C in the dark. Plates were scored for prototrophic growth every 7 days and until 28–35 days of incubation. Positive complementation was indicated by the formation of a dense, aerial growth where mycelia from the tester and the test *nit* mutant had met and formed a prototrophic heterokaryon (**Supplementary Figure S2**). The test *nit* mutant was thus considered vegetatively compatible with the tester strain and was assigned to its VCG.

### Molecular Characterization

The molecular characterization of all the isolates was performed using diagnostic primers of race 1 (Usami et al., 2007; de Jonge et al., 2012) and race 2 (Short et al., 2014), as well as with the diagnostic markers of D and ND pathotypes described for *Vd* infecting olive tree and artichoke (Carder et al., 1994; Mercado-Blanco et al., 2001, 2002, 2003; Collado-Romero et al., 2009). Isolates of the D pathotype of *Vd* are pathogenic on sunflower among other crop species (Jiménez-Díaz et al., 2017), but previous findings by our research group showed that those affecting sunflower are molecularly similar to ND isolates of the fungus that are pathogenic to artichoke or cotton (García-Carneros et al., 2014). Besides, races of *Vd* from sunflower have been determined on the basis of the reaction of particular host genotypes (or differentials) carrying genes of resistance from different sources, but racial characterization by means of molecular markers for race has not been addressed so far. Total genomic DNA from each isolate was purified using the i-Genomic Plant DNA Extraction Minikit (Intron Biotechnology, Sangdaewon-Dong, South Korea) according to the manufacturer’s instructions. Quality and concentration of DNA samples were determined using a Qubit^TM^ 3.0 Fluorometer (Invitrogen^TM^, Carlsbad, CA, United States). Finally, DNA samples were adjusted to a final concentration of 10 ng/μL and stored at -20°C until required.

The primer pairs used for the diagnosis of D and ND pathotypes were: DB19/DB22, DB19/espdef01, INTD2f/INTD2r, INTND2f/INTND2r, INTNDf/INTNDr and INTND2f/INTND3r. Optimized PCR assays were carried out in a final volume of 25 μL containing 0.4 μM each primer, 800 μM dNTPs, 2.5 μL 10x PCR buffer (800 mM tris–HCl, pH 8.3–8.4 at 25°C, 0.2% Tween20 wt/V), 0.75 U Taq-DNA Polymerase (Dominion MBL, Córdoba, Spain), 1.5 mM (DB19/DB22 primers) or 2 mM (rest of primers) MgCl_2_. Amplification conditions were as follows: 4 min denaturation at 94°C; followed by 35 cycles of 1 min denaturation at 94°C, 1 min of annealing at 54°C (DB19/DB22), 62°C (DB19/espdef01), 64°C (INTD2f/INTD2r, INTND2f/INTND2r, INTNDf/INTNDr), 60°C (INTND2f/INTND3r), and 1 min of extension at 72°C; and a final extension step of 6 min at 72°C. Determination of races 1 and/or 2 was conducted using diagnostic primer pairs Tr1/Tr2, VdAve1F/VdAve1R and VdR2F/VdR2R. Optimized PCR assays were carried out in a final volume of 25 μL containing 10 μM each primer, 400 μM dNTPs, 2.5 μL 10x PCR buffer (800 mM tris–HCl, pH 8.3–8.4 at 25°C, 0.2% Tween 20 wt/V), 0.75 U Taq-DNA Polymerase (Dominion MBL, Córdoba, Spain), 3 mM MgCl_2_. The following profiles were set for amplifications: 2 min initial denaturation at 94°C; 35 cycles of 1 min denaturation at 94°C, 1 min annealing at 64°C (Tr1/Tr2 and VdR2F/VdR2R) or 62°C (VdAve1F/VdAve1R), and 1 min of extension at 72°C; and a final extension step of 10 min at 72°C.

All reactions were done in a T1 Thermocycler (Whatman Biometra, Göttingen, Germany). Amplification products were separated by horizontal electrophoresis in 1.5 or 2% agarose gels containing 0.05 μl/ml GoldView Nucleic Acid Stain (SBS Genetech, Beijing, China) and visualized over a UV light source. A 100- to 2,000-bp or 100- to 1,000-bp ladder (Dominion MBL, Cordoba, Spain) was included in the electrophoresis.

A binary matrix based on presence (1) or absence (0) of PCR product was generated. Cluster analysis using the unweighted paired group method with arithmetic averages (UPGMA) algorithm and Jaccard’s similarity coefficient (Jaccard, 1908) were used to classify the isolates and determine genetic similarities among them. Analyses were performed with InfoStat Software^®^ v. 2010 (Di Rienzo et al., 2010).

### Host Range: Pathogenicity of Isolates of *Verticillium dahliae* From Sunflower on Herbaceous Crop Species

An experiment was conducted under greenhouse conditions. Six crop species were inoculated with three isolates of *Vd* from sunflower (VdS0112, VdS0113, and VdS0213) and two from olive tree (one from the D pathotype, VdO0913, and another from the ND pathotype, VdO1113, both of them belonging to the fungal collection of the Laboratory of Field Crop Diseases at the Institute for Sustainable Agriculture, Córdoba, Spain).

Seeds of genetically susceptible artichoke (‘Talpiot’), cotton (‘Avangard’), eggplant (‘Cristal’), lettuce (‘Maravilla de verano’), tomato (‘Manacor’) and sunflower (HA 89 hybrid) were surface-sterilized by surface-sterilized by immersing them in 10% sodium hypochlorite for 10 min, then thoroughly rinsed in deionised water and incubated in the dark at saturation humidity in a germinator at 26 ± 2°C until radicles were 2–5 mm long. Seedlings were then transplanted into vermiculite and incubated in the greenhouse at 15–25°C and photoperiod of 14 h light per day for 1 month. Fertilization was applied weekly using a commercial solution (COMPO Universal Fertilizer) following manufacturer’s recommendation. Then, six plants (replications) of each crop species were uprooted and inoculated by root immersion in conidial suspensions of the *Vd* isolates (10^6^ conidia/ml). Roots of control plants were immersed in deionized water. The experiment was carried out in a completely randomized 6 × 6 factorial design. Plants were incubated for 8 weeks in the greenhouse under the same conditions previously described. Severity of symptoms (SS) in each plant was assessed weekly as a percentage of the foliar tissue affected. Sequential SS values were used to calculate the area under the disease progress curve (AUDPC) by trapezoidal integration method (Campbell and Madden, 1990). The experiment was performed twice and, as no significant differences between the two replicates were found for AUDPC (McIntosh, 1983), data were pooled and analysed using analysis of variance (ANOVA). Mean values of AUDPC were compared using Fisher’s protected least significant difference (LSD) tests (*P* = 0.05). Statistical analyses of data were performed using STATISTIX 10.0 software (Analytical Software, Tallahassee, FL, United States).

### Pathogenic Characterization of *Verticillium dahliae* in Sunflower Genotypes

The pathogenic characterization of *Vd* from sunflower was conducted by means of two phenotyping experiments carried out under greenhouse conditions (20–25°C day and 15–20°C night, with 14 h light). Both experiments were replicated and similar results were obtained. In the first experiment, seven genotypes of sunflower, inbred lines and commercial hybrids with different responses to Verticillium wilt according to previous unpublished data (from field and greenhouse experiments), were independently inoculated with 21 isolates (**Table 1**). Inbred lines were Pioneer 1 and the public lines HA 458, HA 89, and INRA2603. The hybrids included were Pioneer 2, Pioneer 3, and Pioneer 4. Four-week-old plants grown as previously described (experiment of host range in previous subheading) were uprooted and inoculated by immersing the roots in a suspension of 10^6^ conidia per ml for 30 min. Roots of the control plants were immersed in water. Inoculated plants were individually transplanted to 0.75 l pots filled with peat:sand (2:1). Four replications (pots) were used for each genotype and *Vd* isolate. Plants were incubated for 25 days and, at the end of the experiment, VWLM was assessed by means of a Disease Index (DI) that was calculated as: DI = AN × SS, where AN is the percent of affected nodes and SS represents the severity of symptoms according to a 0–5 scale based on chlorosis and necrosis of leaves proposed by Alkher et al. (2009) (0 = no chlorosis or necrosis, 1 = visible chlorosis with <1% necrosis, 2 = up to 40% chlorosis and 1–20% necrosis, 3 = up to 65% chlorosis and 20–35% necrosis, 4 = 100% chlorosis and 35–70% necrosis and 5 = 100% chlorosis and 70–100% necrosis). This scale was used because it represents, with only one value, both the area of the plant with symptoms and the severity of those symptoms. The experiment was performed twice and, as no significant difference between the two replicates was found for DI (McIntosh, 1983), data were pooled and VWLM assessed using ANOVA. Hierarchical cluster analysis with UPGMA algorithm and Euclidean distance was made to classify the isolates into different pathotypes. A principal component analysis (PCA) was also carried out to visualize the distribution of the variability found. Statistical, clustering and PCA analyses were performed with InfoStat Software^®^ v. 2010.

A second phenotyping experiment was established in order to confirm the existence of pathogenic variants and to identify differentials of races of *Vd*. This experiment was performed with inbred lines and a selection of those isolates representing the diversity found after UPGMA analysis in the first experiment. Thus, 12 isolates of *Vd* were inoculated into HA 458, HA 89, INRA2603, and Pioneer 1. The experiment was conducted like experiment 1, with slight modifications. Three-week-old sunflowers were grown and inoculated as described. Aiming at obtaining similar disease data to those occurring under field conditions, sunflowers were transplanted into 3.5 l pots for 6 weeks. At the end of the experiment, VWLM in the plants was evaluated using the DI explained above. The experiment was conducted twice and the data pooled since no significant difference between the two replicates was found for DI (McIntosh, 1983). The DI results were analyzed using ANOVA and, when significant effects were obtained, Fisher’s protected LSD tests (*P* = 0.05) were used for comparisons of genotypes, *Vd* isolates, and their interaction. Statistical analyses of data were performed using InfoStat Software^®^ v. 2010.

## Results

### Genetic Diversity: Determination of Vegetative Compatibility Groups

Of the 36 isolates of *Vd* from sunflower characterized to VCG, 17 were assigned to VCG2B and 6 were assigned to VCG6. The remaining isolates could not be assigned to any VCG. The two isolates from olive tree belonged to VCG1A (VdO0913 isolate, D pathotype), and to VCG2A (VdO1113 isolate, ND pathotype). Surprisingly, VCGs of *Vd* from sunflower were related to their geographical origin. All the isolates from Argentina, France, Italy, and Spain were assigned to VCG2B, while isolates from Bulgaria, Turkey, Romania, and Ukraine were assigned only to VCG6. In this latter group, 13 of the 19 isolates failed to form stable heterokaryons with any of the *nit* mutant testers (**Table 1**).

### Molecular Characterization

With regard to molecular characterization, all the isolates amplified, as expected, the 543- or 526-bp marker specific to *Vd* (DB19/DB22 primers).

When amplified using primer pairs INTD2f/INTD2r (462-bp marker) and DB19/espdef01 (334-bp marker), all the isolates from Argentina and Western Europe showed the 462 (-), 334 (-) pattern. When isolates of *Vd* from Eastern European countries were amplified using the same pairs of primers, they had the following patterns: 462 (+), 334 (+); 462 (-), 334 (+); and 462 (-), 334 (-). This same group of isolates had a single molecular pattern after amplifications with INTNDf/INTNDr (1,163-bp marker), INTND2f/INTND2r (824-bp marker), and INTND2f/INTND3r (688-bp marker): 1,163 (-), 824 (-), 688 (-). Conversely, PCR assays of *Vd* isolates from Argentina and Western European countries using these three pairs of primers amplified either the three markers, only the 688-bp marker, or any combination of two out of the three of them (**Supplementary Table S1**).

When amplified with race-specific primers, all 38 isolates yielded 256-bp amplicons with VdR2F/VdR2R and failed to amplify with Tr1/Tr2 and VdAve1F/VdAve1R (**Supplementary Table S1**). Since no polymorphisms were detected in our *Vd* isolates when using race-specific primers, these data were omitted for the molecular analysis.

**FIGURE 1:**
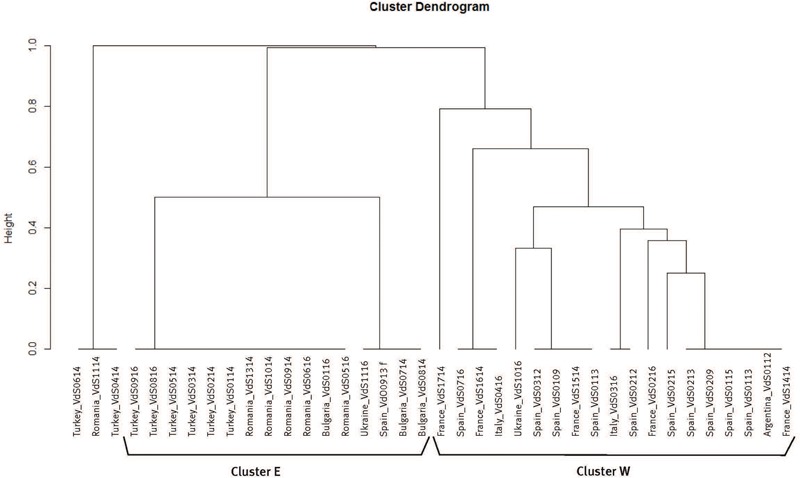
UPGMA dendrogram based on molecular marker data for 38 *Verticillium dahliae* (*Vd*) isolates from sunflower and olive tree.

The dendrogram resulting from the UPGMA analysis of the molecular data set for pathogenic characterization distinguished three well-differentiated clusters among the 38 isolates of *Vd* (**Figure 1**). The first cluster (Cluster E) grouped the 16 isolates collected in countries from Eastern Europe as well as the isolate VdO0913 from olive tree collected in Spain, which shared about a 50% similarity. Moreover, all the isolates grouped in cluster E belonged to an unknown VCG or to VCG6. All isolates of *Vd* from sunflower of Argentina and Western Europe countries, as well as one isolate from Ukraine and the isolate VdO0113 from olive tree of Spain, shared a 21% similarity and were grouped in a second cluster (cluster W) irrespective of their country of origin. Interestingly, all the isolates in cluster W were assigned to VCG2B. Finally, two isolates from Turkey (VdS0614 and VdS0414) and one from Romania (VdS1114) were genetically very distant from the rest of the isolates.

### Host Range: Pathogenicity of Isolates of *Verticillium dahliae* From Sunflower in Herbaceous Crop Species

**FIGURE 2:**
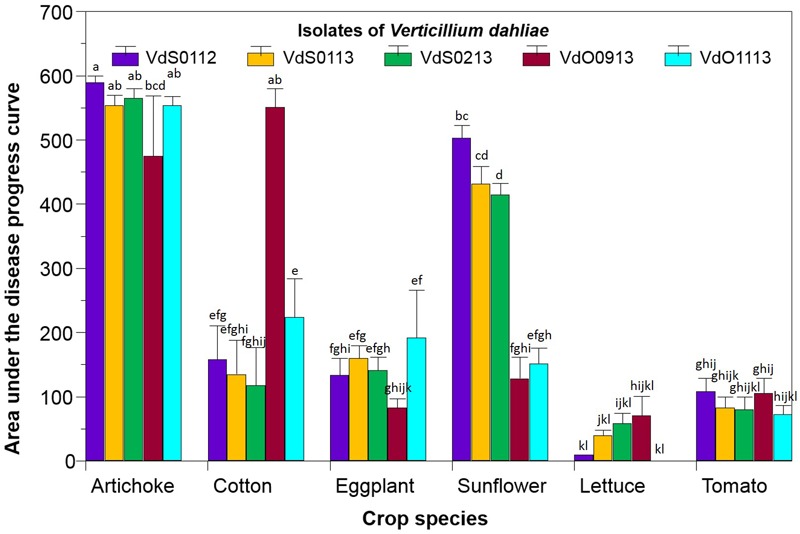
Reaction, expressed as the area under the disease progress curve, of six crop species upon inoculation with three isolates of *Verticillium dahliae* from sunflower (VdS0112, VdS0113, and VdS0213) and two from olive tree (VdO0913 and VdO1113). Bars with the same lower case letters are not significantly different according to the least significant difference test (*P* = 0.05, critical least significant difference value = 86.80).

The disease by *Vd*, expressed as the AUDPC, caused by the three isolates from sunflower and the two from olive tree on artichoke, cotton, eggplant, sunflower, lettuce, and tomato is depicted in **Figure 2**. Data for non-inoculated control plants are not shown since they were zero. Statistical analyses showed that both main factors -crop species and isolate of *Vd*- had a significant impact (*P* < 0.0001) on the disease, but also a significance (*P* < 0.0001) of the crop species × isolate of *Vd* interaction was obtained, indicating that the AUDPC of each crop species was influenced by the particular *Vd* isolate infecting it. Overall, the susceptibility of the crop species studied varied from low in the case of lettuce and tomato (36 and 90 AUDPC across isolates, respectively), to high and very high in that of sunflower (326 AUDPC across isolates) and artichoke (548 AUDPC across isolates), respectively (**Figure 2**). Likewise, the pathogenic ability of *Vd* isolates was dependent on the crop species that they were infecting. None of them were pathogenic to lettuce or tomato, since disease values did not significantly differ from those of the non-inoculated controls. On the contrary, all the isolates were highly pathogenic to artichoke, with AUDPC values ranging from 590 for 112 to 475 for 1333 (**Figure 2**).

The most interesting reactions to *Vd* were those of cotton and sunflower. Cotton was highly susceptible to the isolate VdO0913 (551 AUDPC), and was moderately susceptible to the rest of the isolates (average 159 AUDPC across them). Sunflower was highly susceptible to those isolates recovered from sunflower samples (450 AUDPC averaged across VdS0112, VdS0113, and VdS0213) but not to those from olive tree (140 AUDPC averaged across them). Finally, eggplant displayed moderate susceptibility to VdO1113 isolate (192 AUDPC in comparison to 129 AUDPC averaged across the remaining four isolates).

### Pathogenic Characterization: Identification of Sunflower Genotypes as Differentials of Races of *Verticillium dahliae* From Sunflower

**FIGURE 3:**
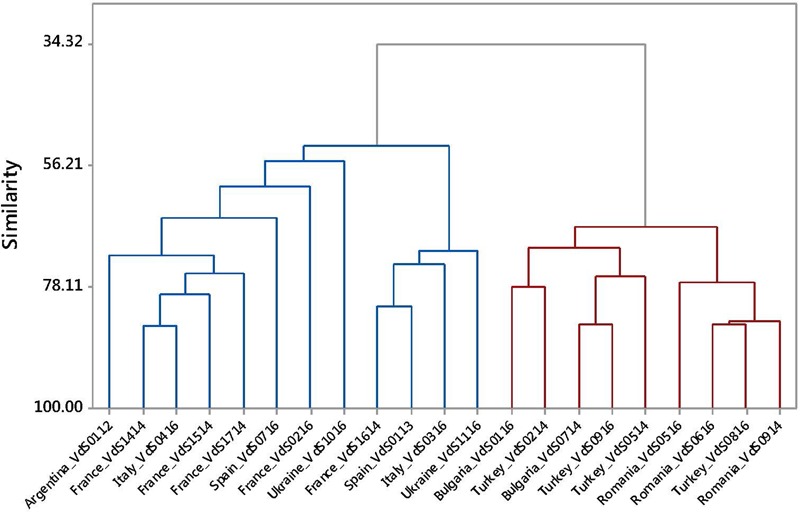
UPGMA dendrogram based on Disease Index values for seven sunflower genotypes inoculated with 21 isolates of *Verticillium dahliae* from sunflower. Blue and red colors are used to indicate Clusters W and E, respectively.

The VdS1116 isolate was pathogenic to HA 458 and Pioneer 3 genotypes but it did not cause any symptom in the rest of the genotypes. All the isolates except VdS1116 induced symptoms in those two genotypes but also in at least one of the others. The UPGMA dendrogram generated with the phenotypic data of experiment 1 shows a first approach to the pathogenic diversity of *Vd* isolates (**Figure 3**). Individual phenotypic information for each isolate is presented in **Supplementary Table S2**. The UPGMA dendrogram shows two big clusters, one including isolates from Western Europe (France, Spain, and Italy), the isolate from Argentina and the two isolates from Ukraine. The other big cluster grouped isolates from Eastern Europe (Turkey, Bulgaria, and Romania). This clustering reflects the reactions of the sunflower genotypes: in the Eastern Europe group, Pioneer 4, HA 89 and Pioneer 1 were the most resistant genotypes, while in the case of isolates from Western Europe, Pioneer 1, Pioneer 2, and INRA2603 were the most resistant ones. In both groups, HA 458 and Pioneer 3 were the most susceptible genotypes. The PCA biplot represented in **Supplementary Figure S3** shows these relationships.

**FIGURE 4:**
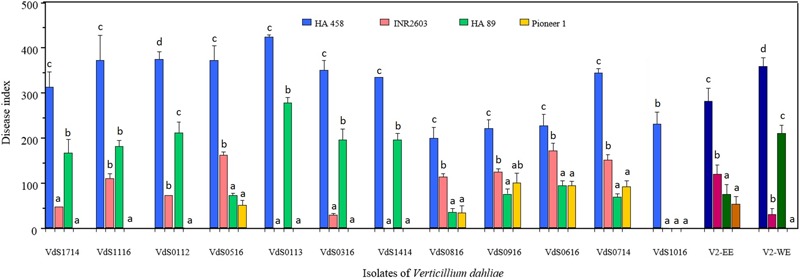
Verticillium wilt and leaf mottle in four sunflower genotypes caused by 12 *Verticillium dahliae* isolates from different geographical origins and expressed by a disease index calculated on the basis of the percent of affected nodes and the severity of symptoms in the plants (see section “Materials and Methods” for details). The last two groups of darker bars represent values averaged for isolates from the east (V2-EE) and the west (V2-WE) of Europe. Bars with the same lower case letters are not significantly different according to the least significant difference test (*P* = 0.05).

**Figure 4** shows the results of experiment 2 in which four sunflower inbred lines (HA 458, HA 89, INRA2603, and Pioneer 1) and 12 isolates representing the diversity observed in experiment 1 were included. Characteristic and differential reactions of sunflower genotypes in experiment 1 were observed again in this experiment. Thus, INRA2603 was resistant to isolates from Western Europe (including those from Argentina and Ukraine) while it was susceptible to isolates from Eastern Europe, and the opposite situation was observed for HA 89 genotype. Pioneer 1 was resistant to both groups of isolates and HA 458 was the one most susceptible to all of them. There were significant differences for DI between genotypes, isolates of *Vd* and their interaction (*P* < 0.0001 for all). In general, genotype × isolate combinations resulting in values of DI between 0 and 100 did not significantly differ from those of the non-inoculated plants so that this criterion of <100 was used to determine resistance interactions. Pioneer 1 line was resistant to all isolates (31 DI averaged across isolates) and HA 458 presented DI values of 200 or higher with all the isolates. The INRA2603 and HA 89 responses depended on the isolates, showing resistance reactions for some of them and susceptible ones for others. As in experiment 1, the VdS1116 isolate was pathogenic only to HA 458 (230 DI). Isolates from Turkey, Bulgaria, and Romania caused higher disease responses than 100 in INRA2603 (144 DI averaged across isolates). This same inbred line was resistant to the rest of the isolates. By contrast, HA 89 was resistant to VdS0714, VdS0516, VdS0616, VdS0816, and VdS0916 (Turkey, Bulgaria, and Romania) (68 DI averaged across isolates) and susceptible to the remaining seven *Vd* isolates (195 DI averaged across them). **Supplementary Figure S1B** shows the symptoms in HA 458, HA 89, INRA2603, and Pioneer 1 inbred lines after inoculation with isolate VdS0316 which corresponds to the typical profile observed for Western Europe *Vd* isolates.

## Discussion

In this study, a low genetic diversity of *Vd* from sunflower was found, with only VCG2B and VCG6 being identified among the isolates. One important finding has been the identification of sunflower as the second host, after pepper (Bhat et al., 2003), in which *Vd* is assigned to VCG6. Moreover, isolates belonging to VCG6 were restricted to Eastern Europe, where we also found a high proportion of *Vd* isolates not assigned to any VCG. This could be related to a genetic diversity of *Vd* that is unidentifiable with the available VCG testers. In contrast, VCG2B was the only group identified for all the *Vd* isolates from Western Europe and Argentina in agreement with previous reports from our group (García-Carneros et al., 2014). The VCG2B has been identified for isolates from *Vd* pathogenic to other herbaceous crops species, such as mint (Douhan and Johnson, 2001), spinach (Iglesias-Garcia et al., 2013), cotton (Göre et al., 2014), watermelon (Dervis et al., 2009a), or eggplant (Dervis et al., 2009b) among others. Our host range results are consistent with host adaptation (Jiménez-Díaz et al., 2017) in *Vd* from sunflower, which was clearly pathogenic to sunflower but not to the other crop species, with the exception of artichoke. Host adaptation means that isolates may be pathogenic on multiple hosts but are usually more virulent on some hosts, typically, but not exclusively, on those from which they were recovered (Bhat and Subbarao, 1999; Douhan and Johnson, 2001; Jiménez-Díaz et al., 2006). Furthermore, the finding that *Vd* from sunflower is pathogenic and highly virulent on artichoke is in agreement with the results of its genetic characterization. All three isolates from sunflower included in the host range study were identified as VCG2B, a frequent VCG in *Vd* from artichoke (Berbegal et al., 2010). Additionally, it was not unexpected to find that isolates of *Vd* from olive tree affected cotton, since cross pathogenicity of the fungus in both crops has been reported (López-Escudero and Mercado-Blanco, 2011). However, according to our results about the molecular and pathogenic diversity of *Vd* in Europe, our conclusions on host range might not be applicable to all isolates of *Vd* in Europe. Host range of *Vd* from sunflower would be better precised if isolates of the fungus from Eastern Europe and VCG different to VCG2B were considered. From the phytopathological point of view, root tissues and plant debris of any crop species infected by *Vd* strains from sunflower can serve as carriers and sources of inoculum. Studies on cross pathogenicity in *Vd* belonging to VCG6 and infecting sunflower and pepper, as well as that of *Vd* belonging to VCG2B and pathogenic to herbaceous hosts such as sunflower, mint, spinach, watermelon, or eggplant are needed to better understand the concern that these crops as farming alternatives can raise for possible severe outbreaks or increased severities of Verticillium wilt.

Molecular markers revealed a haplotype diversity that suggests a clear divergence between *Vd* from the east and west of Europe. An important finding of this study is that molecular differences in *Vd* from sunflower were mostly related to ND and D pathotypes, since all the isolates were race 2. Since *Ave1* was not amplified from any of the haplotypes of *Vd* from sunflower nor from either of the two from olive tree, all of them lack this gene (de Jonge et al., 2012). Hu et al. (2015) found that ND and D isolates of *Vd* from cotton correlated with races 1 and 2. In our research ND and D pathotypes of *Vd* from sunflower were identified irrespective of race. Geographical differences were found instead: the ND pathotype was identified for haplotypes of *Vd* from Western Europe (cluster W) and the D pathotype for haplotypes from Eastern Europe (cluster E). Another interesting outcome of our study is the unexpectedly strong agreement between haplotype clustering and genetic characterization, with only VCG6 identified in cluster E (east of Europe) and VCG2B the only genetic group in haplotypes from cluster W (west of Europe). Although race identification using molecular biological methods is more useful than time-consuming inoculation experiments, the molecular identification of those pathogenic differences in *Vd* from sunflower that are clearly distinguished on the basis of phenotypic data is still not possible. Differential pathogenicity within race 2 has also recently been reported by Usami et al. (2017) for *Vd* from tomato.

Little is known about the pathogenic diversity of *Vd* in sunflower. Understanding this diversity of *Vd* populations in Europe and determining the pathogenic races that are present in the area is an essential and determining requirement for efficient resistance breeding. In this study we found that the new race overcoming the *V_1_* gene into HA 89 (VdS0113 isolate) (García-Ruiz et al., 2014) can be effectively controlled by the resistance in the public line INRA2603. Moreover, INRA2603 frequently presented reverse reactions to the *Vd* isolates to those of HA 89. Overall, those *Vd* isolates effectively controlled by INRA2603 were not controlled by HA 89. This was the case of *Vd* isolates from Argentina, France, Italy, and Spain. On the contrary, INRA2603 was susceptible to isolates from Bulgaria, Turkey, and Romania to which HA 89 was resistant. These results suggest that the nature of the resistance of INRA2603 to *Vd*, and, probably, its associated resistance mechanism/s, is different to that in HA 89. On the other hand, we propose to name these pathogenic races of *Vd* as: (a) V2-EE (“Verticillium race 2 East Europe,” pathogenic on INRA2603 but not on HA 89), (b) V2-WE (“Verticillium race 2 West Europe,” pathogenic on HA 89 but not on INRA2603), and (c) V1 (the race controlled by both HA 89 and INRA2603). Another finding of our study is that race V2-WE of *Vd* is not only present in Spain, but also in Argentina (VdS0112), France (VdS1414 and VdS1714), Italy (VdS0316), and Ukraine (VdS1016). Whether or not race V2-WE has the same pathogenic abilities as isolates of *Vd* overcoming *V_1_* (HA 89) in Argentina (Bertero de Romano and Vázquez, 1982; Galella et al., 2004) and/or in the United States (Gulya, 2007) remains unknown.

The most useful differentials for race characterization of plant pathogens -including those of sunflower- are public inbred lines, since their genetic background is known and they can easily be exchanged between research groups (Molinero-Ruiz et al., 2015). The presence of four pathogenic races has recently been reported in Argentina (Clemente et al., 2017) on the basis of the use of a set of differentials that is not public. Since INRA2603 and HA 89 are public lines and differentially resistant and/or susceptible to *Vd* in Europe, we propose that they should be used as differentials for pathogenic races of *Vd*. Thus, the set for identification of pathogenic races of *Vd* would be: HA 458 (universal susceptible), HA 89 and INRA2603.

## Conclusion

The current study constitutes the first research work focused on the characterization of *Vd* on sunflower in Europe. Its findings provide new insights into *Vd* populations affecting sunflower, a preliminary description of three genotypes to establish a universal set of race differentials like, for example, those in downy mildew – sunflower (Tourvieille de Labrouhe et al., 2000), and have fundamental implications for resistance breeding. First, we found that the *Vd* isolates from sunflower lack the *Ave1* gene and are molecularly distinguished into two different groups: Western Europe and Eastern Europe, their differences being associated with ND and D pathotypes, respectively. Even genetic differences were found between both groups, VCG2B being described in *Vd* from the west of Europe and VCG6 being assigned only to isolates from the east of Europe. With respect to pathogenic characterization of *Vd* from sunflower, and in addition to race V1, races V2-EE and V2-WE were determined according to the sources of resistance that they overcome (HA 89 and INRA2603 inbred lines). Secondly, any search for resistance to *Vd* for European environments of sunflower production should take this diversity into account in order to find donors with a broad resistance that can be effective to both V2-EE and V2-WE races. Otherwise, this pathogenic variability must be properly managed through the development of hybrids with resistance to specific geographical areas (Western and Eastern Europe). This research constitutes a milestone in analyzing the diversity of *Vd* in countries of Europe where sunflowers are grown. Collaborations between public and private sectors similar to that of this work should be advisable in other areas where Verticillium poses a threat to this oil crop.

## Author Contributions

AM-S and LM-R analyzed the data, interpreted the results, conceived and designed the experiments, and contributed materials, equipment, and analysis tools. AM-S, SR, AG-C, SG-F, PM-F, and SC-S conducted the experiments. AM-S, AG-C, PM-F, SC-S, and LM-R wrote the manuscript. All authors reviewed the manuscript and approved the final version.

## Conflict of Interest Statement

The authors declare that the research was conducted in the absence of any commercial or financial relationships that could be construed as a potential conflict of interest.

## Supplementary Material

FIGURE S1 **(A)** Severe infection by *Verticillium dahliae* (*Vd*) in sunflower. **(B)** Symptoms produced by *Vd* isolate VdS0316 in the four sunflower genotypes used for race characterization.

FIGURE S2 Example of the formation of a prototrophic heterokaryon.

FIGURE S3 Principal coordinates analysis of the seven sunflower genotypes used in the pathogenic characterization and the 21 *Verticillium dahliae* isolates used in this study.

TABLE S1 Molecular characterization of isolates of *Verticillium dahliae* (*Vd*) from sunflower using markers diagnostic of defoliating (D) and not defoliating (ND) pathotypes and of races 1 and 2 of the fungal species.

TABLE S2 Phenotypic data of experiment 1 of the pathogenic characterization of 21 *Verticillium dahliae* isolates inoculated on seven sunflower genotypes.

## References

[B1] Alexander L. J. (1962). Susceptibility of certain Verticillium-resistant tomato varieties to an Ohio isolate of the pathogen. *Phytopathology* 52 998–1000.

[B2] Alkher H. El Hadrami A. Rashid K. Y. Adam L. R. Daayf F. (2009). Cross-Pathogenicity of *Verticillium dahliae* between potato and sunflower. *Eur. J. Plant Pathol.* 124 505–519. 10.1007/s10658-009-9437-z

[B3] Bejarano-Alcázar J. Blanco-López M. A. Melero-Vara J. M. Jiménez-Díaz R. M. (1996). Etiology, importance and distribution of Verticillium wilt of cotton in southern Spain. *Plant Dis.* 80 1233–1238. 10.1094/PD-80-1233

[B4] Berbegal M. Ortega A. Jiménez-Gasco M. M. Olivares-García C. Jiménez-Díaz R. M. Armengol J. (2010). Genetic diversity and host range of *Verticillium dahliae* isolates from artichoke and other vegetable crops in Spain. *Plant Dis.* 94 396–404. 10.1094/PDIS-94-4-0396 30754522

[B5] Bertero de Romano A. B. Vázquez A. (1982). “A new race of *Verticillium dahlia* Kleb,” in *Proceedings of the 10th International Sunflower Conference* Toowoomba, QLD 177–178.

[B6] Bhat R. Smith R. F. Koike S. T. Wu B. M. Subbarao K. V. (2003). Characterization of *Verticillium dahliae* isolates and wilt epidemics of pepper. *Plant Dis.* 87 789–797. 10.1094/PDIS.2003.87.7.789 30812888

[B7] Bhat R. G. Subbarao K. V. (1999). Host range specificity in *Verticillium dahliae*. *Phytopathology* 89 1218–1225. 10.1094/PHYTO.1999.89.12.1218 18944648

[B8] Campbell C. L. Madden L. V. (1990). *Introduction to Plant Disease Epidemiology.* New York, NY: John Wiley & Sons.

[B9] Carder J. H. Morton A. Tabrett A. M. Barbara D. J. (1994). “Detection and differentiation by PCR of subspecific groups within two Verticillium species causing vascular wilts in herbaceous hosts,” in *Modern Assays for Plant Pathogenic Fungi* eds Schots A. Oliver F. M. Dewey R. (Oxford: CAB International) 91–97.

[B10] Clemente G. E. Bazzalo M. E. Escande A. R. (2017). New variants of *Verticillium dahliae* causing sunflower leaf mottle and wilt in Argentina. *J. Plant Pathol.* 99 445–451. 10.4454/jpp.v99i2.3875

[B11] Collado-Romero M. Berbegal M. Jiménez-Díaz R. M. Armengol J. Mercado-Blanco J. (2009). A PCR-based “molecular tool box” for in planta differential detection of *Verticillium dahliae* vegetative compatibility groups infecting artichoke. *Plant Pathol.* 58 515–526. 10.1111/j.1365-3059.2008.01981.x

[B12] Collado-Romero M. Mercado-Blanco J. Olivares-García C. Jiménez-Díaz R. M. (2008). Phylogenetic analysis of *Verticillium dahliae* vegetative compatibility groups. *Phytopathology* 98 1019–1028. 10.1094/PHYTO-98-9-1019 18943740

[B13] Collado-Romero M. Mercado-Blanco J. Olivares-García C. Valverde-Corredor A. Jiménez-Díaz R. M. (2006). Molecular variability within and among *Verticillium dahliae* vegetative compatibility groups determined by fluorescent AFLP and PCR markers. *Phytopathology* 96 485–495. 10.1094/PHYTO-96-0485 18944308

[B14] Correll J. C. Klittich C. J. R. Leslie J. F. (1987). Nitrate nonutilizing mutants of *Fusarium oxysporum* and their use in vegetative compatibility tests. *Phytopathology* 77 1640–1646. 10.1094/Phyto-77-1640

[B15] de Jonge R. van Esse H. P. Maruthachalam K. Bolton M. D. Santhanam P. Saber M. K. (2012). Tomato immune receptor Ve1 recognizes effector of multiple fungal pathogens uncovered by genome and RNA sequencing. *Proc. Natl. Acad. Sci. U.S.A.* 109 5110–5115. 10.1073/pnas.1119623109 22416119 PMC3323992

[B16] Debaeke P. Bedoussac L. Bonnet C. Bret-Mestries E. Seassau C. Gavaland A. (2017). Sunflower crop: environmental-friendly and agroecological. *OCL* 24:D304 10.1051/ocl/2017020

[B17] Dervis S. Kurt S. Soylu S. Erten L. Soylu E. M. Yildiz M. (2008). Vegetative compatibility groups of *Verticillium dahliae* from cotton in the Southeastern Anatolia Region of Turkey. *Phytoparasitica* 36 74–83. 10.1007/BF02980750

[B18] Dervis S. Mercado-Blanco J. Erten L. Valverde-Corredor A. Pérez-Artés E. (2010). Verticillium wilt of olive in Turkey: a survey on disease importance, pathogen diversity and susceptibility of relevant olive cultivars. *Eur. J. Plant Pathol.* 127 287–301. 10.1007/s10658-010-9595-z

[B19] Dervis S. Yetisir H. Tok F. M. Kurt S. Karaca F. (2009a). Vegetative compatibility groups and pathogenicity of *Verticillium dahliae* isolates from watermelon in Turkey. *Afr. J. Agric. Res.* 4 1268–1275. 10.1046/j.1365-3059.1998.00273.x

[B20] Dervis S. Yetisir H. Yildirim H. Tok F. M. Kurt S. Karaca F. (2009b). Genetic and pathogenic characterization of *Verticillium dahliae* isolates from eggplant in Turkey. *Phytoparasitica* 37 467–476. 10.1007/s12600-009-0061-4

[B21] Di Rienzo J. A. Casanoves F. Balzarini M. G. Gonzalez L. Tablada M. Robledo C. W. (2010). *InfoStat (Version 2010).* Cordoba: Unidad Nacional de Cordoba.

[B22] Douhan L. I. Johnson D. A. (2001). Vegetative compatibility and pathogenicity of *Verticillium dahliae* from spearmint and peppermint. *Plant Dis.* 85 297–302. 10.1094/PDIS.2001.85.3.297 30832046

[B23] Dung J. K. S. Peever T. L. Johnson D. A. (2013). *Verticillium dahliae* populations from mint and potato are genetically divergent with predominant haplotypes. *Phytopathology* 103 445–459. 10.1094/PHYTO-06-12-0133-R 23113547

[B24] El-Bebany A. F. Alkher H. Lorne R. A. Daayf F. (2013). Vegetative compatibility of *Verticillium dahliae* isolates from potato and sunflower using nitrate non-utilizing (nit) mutants and PCR-based approaches. *Can. J. Plant Pathol.* 35 1–9. 10.1080/07060661.2012.702128

[B25] Fick G. N. Zimmer D. E. (1974). Monogenic resistance to Verticillium wilt in sunflowers. *Crop Sci.* 14:895 10.2135/cropsci1974.0011183X001400060037x

[B26] Galella M. T. Bazzalo M. E. León A. (2004). “Compared pathogenicity of *Verticillium dahlia* isolates from Argentine and the USA,” in *Proceedings of the 16th International Sunflower Conference* Fargo, ND 177–180.

[B27] Galella M. T. Bazzalo M. E. Morata M. Cimmino C. Kaspar M. Grondona M. (2012). “Pyramiding QTLs for *Verticillium dahliae* resistance,” in *Proceedings of the 18th International Sunflower Conference* Mar del Plata 219–224.

[B28] García-Carneros A. B. García-Ruiz R. Molinero-Ruiz L. (2014). Genetic and molecular approach to *Verticillium dahliae* infecting sunflower. *Helia* 37 205–214. 10.1515/helia-2014-0014

[B29] García-Ruiz R. García-Carneros A. B. Molinero-Ruiz L. (2014). A new race of *Verticillium dahliae* causing leaf mottle of sunflower in Europe. *Plant Dis.* 98:1435 10.1094/PDIS-04-14-0360-PDN 30703974

[B30] Göre M. E. Erdoğan O. Caner O. K. Aydın M. H. Berk S. (2014). VCG diversity and virulence of *Verticillium dahliae* from commercially available cotton seed lots in Turkey. *Eur. J. Plant Pathol.* 140 689–699. 10.1007/s10658-014-0500-z

[B31] Gulya T. (2007). New strain of *Verticillium dahliae* in North America. *Helia* 30 115–120. 10.2298/HEL0747115G

[B32] Gulya T. J. Rashid K. Y. Marisevic S. M. (1997). “Sunflower diseases,” in *Sunflower Technology and Production* ed. Schneiter A. A. (Madison, WI: ASA) 263–380.

[B33] Harveson R. M. Markell S. G. (2016). “Verticillium wilt,” in *Compendium of Sunflower Diseases* eds Harveson R. M. Markell S. G. Block C. C. Gulya T. J. (St. Paul, MN: The American Phytopathological Society) 59–61.

[B34] Hayes R. J. McHale L. K. Vallad G. E. Truco M. J. Michelmore R. W. Klosterman S. J. (2011). The inheritance of resistance to Verticillium wilt caused by race 1 isolates of *Verticillium dahliae* in the lettuce cultivar La Brillante. *Theor. Appl. Genet.* 123 509–517. 10.1007/s00122-011-1603-y 21567237

[B35] Hayes R. J. Vallad G. E. Qin Q. M. Grube R. C. Subbarao K. V. (2007). Variation for resistance to Verticillium wilt in lettuce (*Lactuca sativa* L.). *Plant Dis.* 91 439–445. 10.1094/PDIS-91-4-0439 30781187

[B36] Hu X. P. Gurung S. Short D. P. G. Sandoya G. V. Shang W. J. Hayes R. J. (2015). Nondefoliating and defoliating strains from cotton correlate with races 1 and 2 of *Verticillium dahliae*. *Plant Dis.* 99 1713–1720. 10.1094/PDIS-03-15-0261-RE 30699524

[B37] Iglesias-Garcia A. M. Villarroel-Zeballos M. I. Feng C. du Toit L. J. Correll J. C. (2013). Pathogenicity, virulence, and vegetative compatibility grouping of Verticillium isolates from spinach seed. *Plant Dis.* 97 1457–1469. 10.1094/PDIS-01-13-0016-RE 30708458

[B38] Jaccard P. (1908). Nouvelles recherches Sur la distribution florale. *Bull. Soc. Vaud. Sci. Nat.* 44 223–270.

[B39] Jiménez-Díaz R. M. Mercado-Blanco J. Olivares-García C. Collado-Romero M. Bejarano-Alcázar J. Rodríguez-Jurado D. (2006). Genetic and virulence diversity in *Verticillium dahliae* populations infecting artichoke in eastern-central Spain. *Phytopathology* 96 288–298. 10.1094/PHYTO-96-0288 18944444

[B40] Jiménez-Díaz R. M. Olivares-García C. Landa B. B. Jiménez-Gasco M. M. Navas-Cortés J. A. (2011). Region-wide analysis of genetic diversity in *Verticillium dahliae* populations infecting olive in southern Spain and agricultural factors influencing the distribution and prevalence of vegetative compatibility groups and pathotypes. *Phytopathology* 101 304–315. 10.1094/PHYTO-07-10-0176 20942654

[B41] Jiménez-Díaz R. M. Olivares-García C. Trapero-Casas J. L. Jiménez-Gasco M. M. Navas-Cortés J. A. Landa B. B. (2017). Variation of pathotypes and races and their correlations with clonal lineages in *Verticillium dahliae*. *Plant Pathol.* 66 651–666. 10.1111/ppa.12611

[B42] Korolev N. Katan T. (1997). Improved medium for selecting nitrate nonutilizing (nit) mutants of *Verticillium dahliae*. *Phytopathology* 87 1067–1070. 10.1094/PHYTO.1997.87.10.1067 18945042

[B43] Korolev N. Katan T. Katan J. (2009). “Physiological races and vegetative compatibility groups among *Verticillium dahliae* isolates from tomato in Israel,” in *Proceedings of the 2nd International Symposium on Tomato Diseases* Kusadasi 57–64. 10.17660/ActaHortic.2009.808.7

[B44] Korolev N. Pérez-Artés E. Mercado-Blanco J. Bejarano-Alcázar J. Rodríguez-Jurado D. Jiménez-Díaz R. (2008). Vegetative compatibility of cotton defoliating *Verticillium dahliae* in Israel and its pathogenicity to various hosts. *Eur. J. Plant Pathol.* 122 603–617. 10.1007/s10658-008-9330-1

[B45] López-Escudero F. J. Mercado-Blanco J. (2011). Verticillium wilt of olive: a case study to implement an integrated strategy to control a soil-borne pathogen. *Plant Soil* 344 1–50. 10.1007/s11104-010-0629-2

[B46] Maruthachalam K. Atallah Z. K. Vallad G. E. Klosterman S. J. Hayes R. J. Davis R. M. (2010). Molecular variation among isolates of *Verticillium dahliae* and polymerase chain reaction based differentiation of races. *Phytopathology* 100 1222–1230. 10.1094/PHYTO-04-10-0122 20698756

[B47] McIntosh M. S. (1983). Analysis of combined experiments. *Agron. J.* 75 153–155. 10.2134/agronj1983.00021962007500010041x

[B48] Mercado-Blanco J. Rodríguez-Jurado D. Parrilla-Araujo S. Jiménez-Díaz R. M. (2003). Simultaneous detection of the defoliating and nondefoliating *Verticillium dahliae* pathotypes in infected olive plants by duplex, nested polymerase chain reaction. *Plant Dis.* 87 1487–1494. 10.1094/PDIS.2003.87.12.1487 30812391

[B49] Mercado-Blanco J. Rodríguez-Jurado D. Pérez-Artés E. Jiménez-Díaz R. M. (2001). Detection of the nondefoliating pathotype of *Verticillium dahliae* in infected olive plants by nested PCR. *Plant Pathol.* 50 609–619. 10.1046/j.1365-3059.2001.00601.x 30812391

[B50] Mercado-Blanco J. Rodríguez-Jurado D. Pérez-Artés E. Jiménez-Díaz R. M. (2002). Detection of the defoliating pathotype of *Verticillium dahliae* in infected olive plants by nested-PCR. *Eur. J. Plant Pathol.* 108 1–13. 10.1023/A:1013994827836 30812391

[B51] Molinero-Ruiz L. Delavault P. Pérez-Vich B. Pacureanu-Joita M. Bulos M. Altieri E. (2015). History of the race structure of *Orobanche cumana* and the breeding of sunflower for resistance to the parasitic weed: a review. *Span. J. Agric. Res.* 13:e10R01 10.5424/sjar/2015134-8080

[B52] Navas-Cortés J. A. Olivares C. Trapero-Casas J. L. Landa B. B. Jiménez-Gasco M. M. Jiménez-Díaz R. M. (2009). “The influence of agronomic factors on prevalence and distribution of *Verticillium dahliae* vegetative compatibility groups and pathotypes infecting olive in Andalusia, southern Spain,” in *Proceedings of the Abstracts Book 10th International Verticillium Symposium* de Corfu 79.

[B53] Pegg G. F. Brady B. L. (2002). *Verticillium Wilts.* Wallingford: CAB International 10.1079/9780851995298.0000

[B54] Pérez-Artés E. García-Pedrajas M. D. Bejarano-Alcázar J. Jiménez Díaz R. M. (2000). Differentiation of cotton-defoliating and nondefoliating pathotypes of *Verticillium dahliae* by RAPD and specific PCR analyses. *Eur. J. Plant Pathol.* 106 507–517. 10.1094/PHYTO-98-2-0167 18943193

[B55] Putt E. D. (1958). Note on resistance of sunflowers to leaf mottle disease. *Can. J. Plant Sci.* 38 274–276. 10.4141/cjps58-044

[B56] Putt E. D. (1964). Breeding behavior of resistance to leaf mottle disease or Verticillium in sunflowers. *Crop Sci.* 4 177–179. 10.2135/cropsci1964.0011183X000400020016x

[B57] Radi S. A. Gulya T. J. (2007). “Sources of resistance to a new strain of *Verticillium dahliae* on sunflower in North America-2006 ” in *Proceedings of the 29th Sunflower Research Workshop* Bismarck, ND 10–11.

[B58] Rodríguez-Jurado D. Blanco-López M. A. Rapoport H. Jiménez-Díaz R. M. (1993). Present status of Verticillium wilt of olive in Andalucía (Southern Spain). *Bull. OEPP EPPO Bull.* 23 513–516. 10.1111/j.1365-2338.1993.tb01362.x

[B59] Sandoya G. V. Gurung S. Short D. P. Subbarao K. V. Michelmore R. W. Hayes R. J. (2017). Genetics of resistance in lettuce to races 1 and 2 of *Verticillium dahliae* from different host species. *Euphytica* 213:20. 10.1007/s10681-016-1813-0 24502204

[B60] Short D. P. G. Gurung S. Maruthachalam K. Atallah Z. K. Subbarao K. V. (2014). Verticillium dahliae race 2-specific PCR reveals a high frequency of race 2 strains in commercial spinach seed lots and delineates race structure. *Phytopathology* 104 779–785. 10.1094/PHYTO-09-13-0253-R 24502204

[B61] Strausbaugh C. A. Eujayl I. A. Martin F. N. (2016). Pathogenicity, vegetative compatibility and genetic diversity of *Verticillium dahliae* isolates from sugar beet. *Can. J. Plant Pathol.* 38 492–505. 10.1080/07060661.2016.1260639

[B62] Tourvieille de Labrouhe D. Gulya T. J. Masirevic S. Penaud A. Rashid K. Y. Virányi F. (2000). “New nomenclature of races of *Plasmopara halstedii* sunflower downy mildew,” in *Proceedings of the 15th International Sunflower Conference* (Toulouse: International Sunflower Association) 61–66.

[B63] Usami T. Ishigaki S. Takashina H. Matsubara Y. Amemiya Y. (2007). Cloning of DNA fragments specific to the pathotype and race of *Verticillium dahliae*. *J. Gen. Plant Pathol.* 73 89–95. 10.1007/s10327-006-0334-4

[B64] Usami T. Momma N. Kikuchi S. Watanabe H. Hayashi A. Mizukawa M. (2017). Race 2 of *Verticillium dahliae* infecting tomato in Japan can be split into two races with differential pathogenicity on resistant rootstocks. *Plant Pathol.* 66 230–238. 10.1111/ppa.12576

[B65] Vallad G. E. Qin Q. M. Grube R. Hayes R. J. Subbarao K. V. (2006). Characterization of race-specific interactions among isolates of *Verticillium dahliae* pathogenic on lettuce. *Phytopathology* 96 1380–1387. 10.1094/PHYTO-96-1380 18943671

