# Supplementary figures and images for: Genetics, Host Range, and Molecular and Pathogenic Characterization of Verticillium dahliae From Sunflower Reveal Two Differentiated Groups in Europe
Source: Front Plant Sci. 2018 Mar 9;9:288. doi: 10.3389/fpls.2018.00288 (PMC5855324; doi:10.3389/fpls.2018.00288)

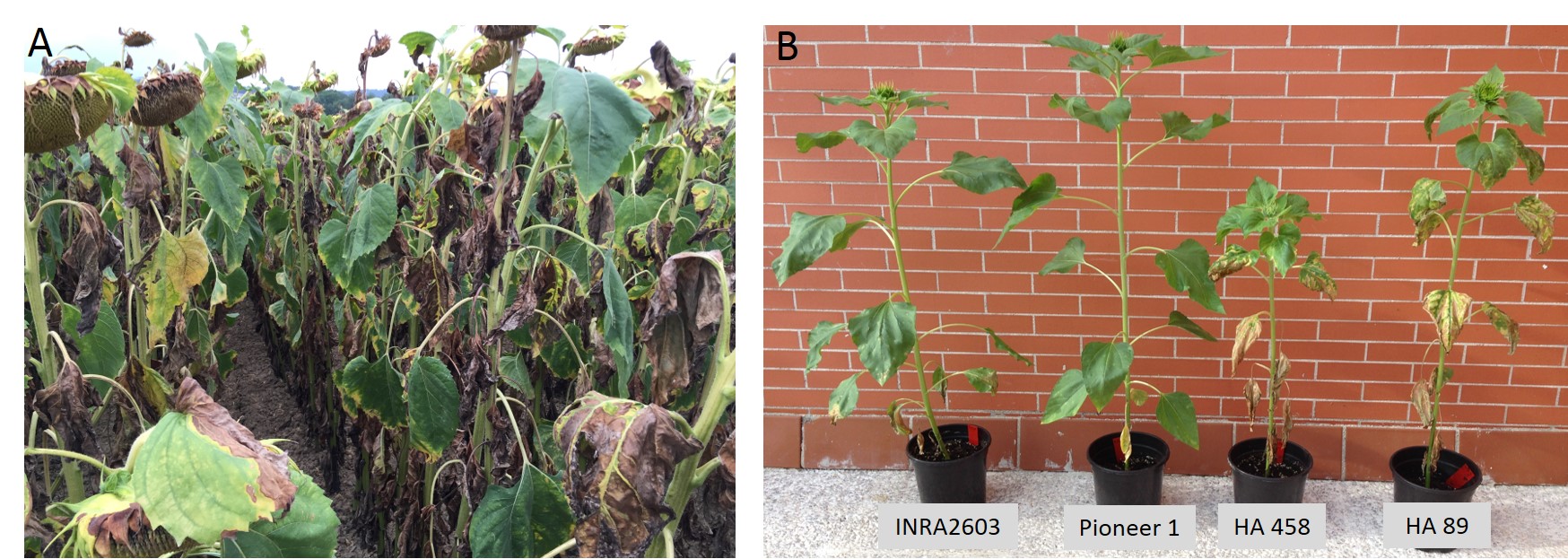

Supplement: FIGURE S1 — (A) Severe infection by Verticillium dahliae (Vd) in sunflower. (B) Symptoms produced by Vd isolate VdS0316 in the four sunflower genotypes used for race characterization. [file Image_1.JPEG]

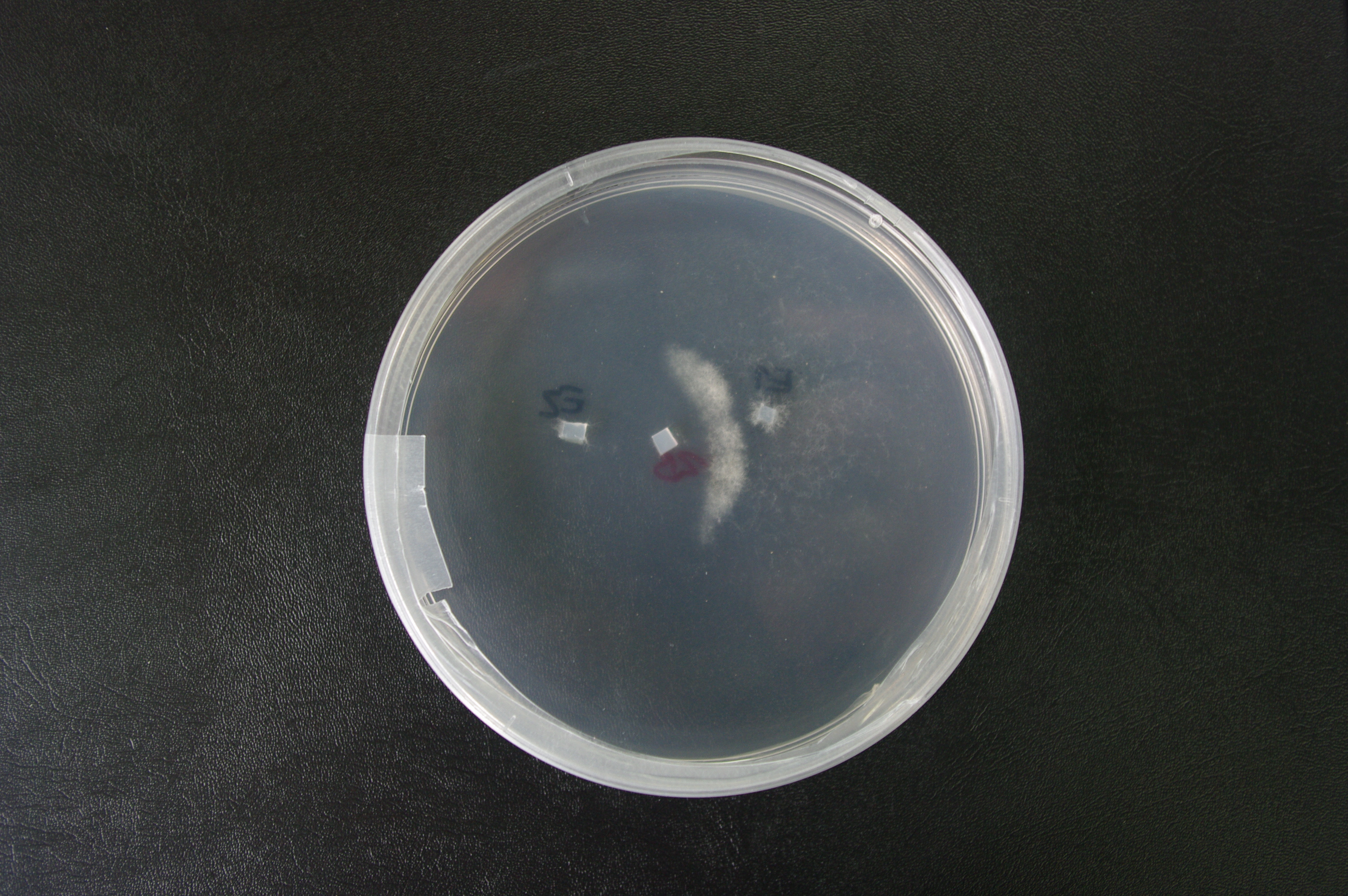

Supplement: FIGURE S2 — Example of the formation of a prototrophic heterokaryon. [file Image_2.JPEG]

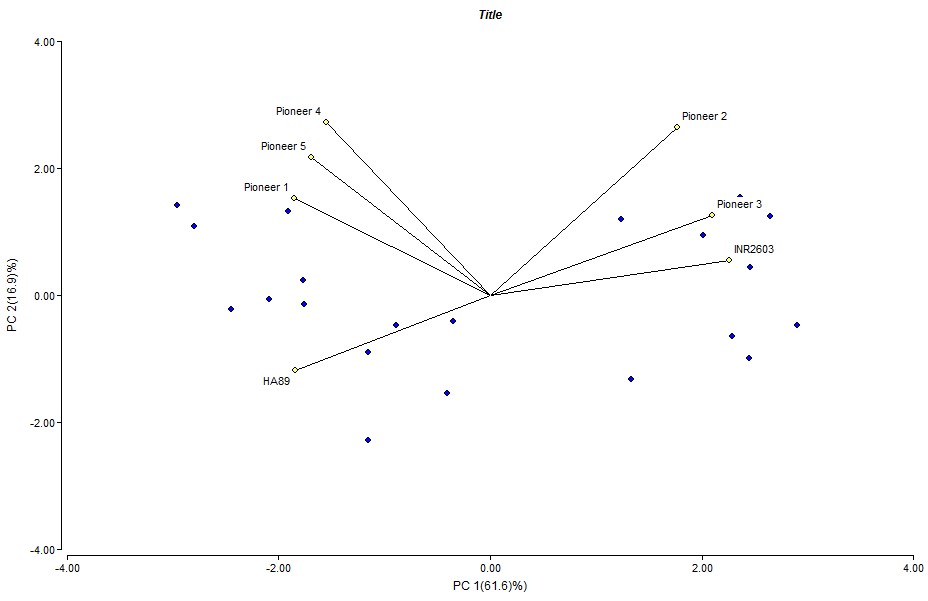

Supplement: FIGURE S3 — Principal coordinates analysis of the seven sunflower genotypes used in the pathogenic characterization and the 21 Verticillium dahliae isolates used in this study. [file Image_3.JPEG]
