# Supplementary material for: Genetics, Host Range, and Molecular and Pathogenic Characterization of Verticillium dahliae From Sunflower Reveal Two Differentiated Groups in Europe
Source: Front Plant Sci. 2018 Mar 9;9:288. doi: 10.3389/fpls.2018.00288 (PMC5855324; doi:10.3389/fpls.2018.00288)
Supplement: TABLE S1 — Molecular characterization of isolates of Verticillium dahliae (Vd) from sunflower using markers diagnostic of defoliating (D) and not defoliating (ND) pathotypes and of races 1 and 2 of the fungal species. [file Table_1.DOCX]

**Supplementary Table 1.** Markers diagnostic of pathotype (D or ND) and race (1 or 2) of *Verticillium dahliae* used in the molecular characterization of isolates from sunflower

| Country of origin |  | Molecular markers (bp) ^a^ | | | | | | | | |
| --- | --- | --- | --- | --- | --- | --- | --- | --- | --- | --- |
|  | Isolate | 526/543 ^a^ | 462 ^b^ | 334 | 1,163 ^c^ | 824 | 688 | 600 ^d^ | 582 | 256 ^e^ |
| Argentina | VdS0112 | + | - | - | + | + | + | - | - | + |
|  |  |  |  |  |  |  |  |  |  |  |
| Bulgaria | VdS0714 | + | + | + | - | - | - | - | - | + |
|  | VdS0814 | + | + | + | - | - | - | - | - | + |
|  | VdS0116 | + | - | + | - | - | - | - | - | + |
|  |  |  |  |  |  |  |  |  |  |  |
| France | VdS1414 | + | - | - | + | + | + | - | - | + |
|  | VdS1514 | + | - | - | + | - | + | - | - | + |
|  | VdS1614 | + | - | - | - | - | + | - | - | + |
|  | VdS1714 | + | - | - | - | + | - | - | - | + |
|  | VdS0216 | + | - | - | - | + | + | - | - | + |
|  |  |  |  |  |  |  |  |  |  |  |
| Italy | VdS0316 | + | - | - | + | + | - | - | - | + |
|  | VdS0416 | + | - | - | - | - | + | - | - | + |
|  |  |  |  |  |  |  |  |  |  |  |
| Romania | VdS0914 | + | - | + | - | - | - | - | - | + |
|  | VdS1014 | + | - | + | - | - | - | - | - | + |
|  | VdS1114 | + | - | - | - | - | - | - | - | + |
|  | VdS1314 | + | - | + | - | - | - | - | - | + |
|  | VdS0516 | + | - | + | - | - | - | - | - | + |
|  | VdS0616 | + | - | + | - | - | - | - | - | + |
|  |  |  |  |  |  |  |  |  |  |  |
| Spain | VdS0109 | + | - | - | + | - | + | - | - | + |
|  | VdS0209 | + | - | - | + | + | + | - | - | + |
|  | VdS0212 | + | - | - | + | + | - | - | - | + |
|  | VdS0312 | + | - | - | + | - | + | - | - | + |
|  | VdS0113 | + | - | - | + | + | + | - | - | + |
|  | VdS0213 | + | - | - | + | + | + | - | - | + |
|  | VdO0913 ^f^ | + | + | + | - | - | - | - | - | + |
|  | VdO1113 | + | - | - | + | - | + | - | - | + |
|  | VdS0115 | + | - | - | + | + | + | - | - | + |
|  | VdS0215 | + | + | - | + | + | + | - | - | + |
|  | VdS0716 | + | - | - | - | - | + | - | - | + |
|  |  |  |  |  |  |  |  |  |  |  |
| Turkey | VdS0114 | + | - | + | - | - | - | - | - | + |
|  | VdS0214 | + | - | + | - | - | - | - | - | + |
|  | VdS0314 | + | - | + | - | - | - | - | - | + |
|  | VdS0414 | + | - | - | - | - | - | - | - | + |
|  | VdS0514 | + | - | + | - | - | - | - | - | + |
|  | VdS0614 | + | - | - | - | - | - | - | - | + |
|  | VdS0816 | + | - | + | - | - | - | - | - | + |
|  | VdS0916 | + | - | + | - | - | - | - | - | + |
|  |  |  |  |  |  |  |  |  |  |  |
| Ukraine | VdS1016 | + | + | - | + | - | + | - | - | + |
|  | VdS1116 | + | + | + | - | - | - | - | - | + |

^a^ Polymorphic sequence specific of *V. dahliae* (Collins et al., 2005).

^b^ Sequences of 462 and 334 bp are diagnostic of D pathotype (Mercado-Blanco et al., 2002; 2003).

^c^ Sequences of 1,163; 824 and 688 bp are diagnostic of ND pathotypes (Mercado-Blanco et al., 2001; Collado-Romero et al., 2009).

^d^ Sequences of 582 and 600 bp are diagnostic of race 1 (de Jonge et al., 2012; Usami et al., 2007).

^e^ Sequence specific of *V. dahliae* race 2 (Short et al., 2014).

^f^ Isolates VdO0913 (D pathotype) and VdO1113 (ND pathotype) of *V. dahliae* from olive tree were included for comparison.
